# Membrane Processing and Steady-State Regulation of the Alternative Peroxisomal Import Receptor Pex9p

**DOI:** 10.3389/fcell.2020.566321

**Published:** 2020-10-22

**Authors:** Markus Rudowitz, Ralf Erdmann, Wolfgang Schliebs

**Affiliations:** Department of Systems Biochemistry, Institute of Biochemistry and Pathobiochemistry, Ruhr-University Bochum, Bochum, Germany

**Keywords:** peroxisome, protein import, Pex5p, PTS1 receptor cycle, Pex9p degradation

## Abstract

Import of peroxisomal matrix proteins with a type 1 peroxisomal targeting signal (PTS1) in *Saccharomyces cerevisiae* is facilitated by cytosolic import receptors Pex5p and Pex9p. While Pex5p has a broad specificity for all PTS1 proteins independent of the growth conditions, Pex9p is only expressed in fatty-acid containing media to mediate peroxisomal import of the two malate synthases, Mls1p and Mls2p, as well as the glutathione transferase Gto1p. Pex5p-cargo complexes dock at the peroxisomal membrane, translocate their cargo-protein via a transient pore and are recycled into the cytosol for a further round of import. The processing of Pex5p has been shown to require a complex network of interactions with other membrane-bound peroxins, as well as decoration with ubiquitin as signal for its ATP-dependent release and recycling. Here, we show that the alternative receptor Pex9p requires the same set of interacting peroxins to mediate peroxisomal import of Mls1p. However, while Pex5p is rather stable, Pex9p is rapidly degraded during its normal life cycle. The steady-state regulation of Pex9p, combining oleate-induced expression with high turnover rates resembles that of Pex18p, one of the two co-receptors of the PTS2-dependent targeting pathway into peroxisomes. Both Pex9p- and Pex18p-dependent import routes serve the fast metabolic adaptation to changes of carbon sources in baker’s yeast. By sequence similarities, we identified another Pex9p homolog in the human pathogenic fungus *Candida glabrata*, in which similar metabolic reprogramming strategies are crucial for survival of the pathogen.

## Introduction

Peroxisomes are ubiquitous cell organelles with variable content of enzymes depending on cell types and environmental or developmental conditions. Peroxisomal enzymes are posttranslationally imported as fully folded and oligomerized proteins and assisted by cytosolic receptor proteins. Most peroxisomal matrix proteins harbor a C-terminal peroxisomal targeting signal type 1 (PTS1), which is recognized by the PTS1-receptor Pex5p (Gould et al., 1989; Van der Leij et al., 1993). Several matrix enzymes contain a PTS2 sequence located near the N-terminus (Glover et al., 1994). PTS2-containing proteins are targeted to peroxisomes by the receptor Pex7p in conjunction with co-factors, like Pex18p or Pex21p in the yeast *Saccharomyces cerevisiae* (Marzioch et al., 1994; Zhang and Lazarow, 1996; Purdue et al., 1998; Kunze et al., 2011).

Upon interaction between the cytosolic receptor-cargo complexes with the docking complex at the peroxisomal membrane, Pex5p or Pex18p associate Pex14p to assemble the respective protein conducting channels of the PTS1- and PTS2-pathway (Albertini et al., 1997; Bottger et al., 2000; Meinecke et al., 2010; Montilla-Martinez et al., 2015). After cargo-release, Pex5p and Pex18p are released into the cytosol again to allow another round of import. The recycling process requires activities of other membrane bound peroxins, ubiquitin-labeling of the receptor as well as ATP-hydrolysis. The releasing process is initiated by cysteine-dependent monoubiquitination at the N-termini of Pex5p or Pex18p (Platta et al., 2007; El Magraoui et al., 2013; Schwerter et al., 2018). In the cytosol, several ways exist to remove the thioether-linked ubiquitin from the receptor (Grou et al., 2009; Debelyy et al., 2011). While the non-labeled receptor escapes proteasomal degradation, some poorly understood quality control pathways can lead to lysine-conjugated polyubiquitination of the membrane-bound receptor, leading to rapid degradation by the proteasome (Platta et al., 2005).

The set of membrane bound peroxins enabling the cycling of import receptors is well conserved between yeast and other organisms. However, yeast cells use differentially expressed receptors, like the PTS2 co-receptors Pex18p and Pex21p (Purdue et al., 1998; Effelsberg et al., 2015, 2016) to ensure efficacy of matrix protein import under rapid changing environmental conditions. In the PTS1-pathway, the function of Pex5p in cargo recognition is complemented by the recently identified oleate-inducible paralog Pex9p (Effelsberg et al., 2016; Yifrach et al., 2016). Some of the structural features of Pex5p, like the PTS1-binding TPR-domains and Pex14p-binding motifs, are conserved in Pex9p (Effelsberg et al., 2016). Despite these similarities, only three PTS1-cargo proteins, the malate synthase isoenzymes Mls1p and Mls2p, and the omega-class glutathione transferase Gto1p, have been identified to be imported with the help of Pex9p (Effelsberg et al., 2016; Yifrach et al., 2016). Pex9p is expressed under fatty acid induced growth conditions but repressed in glucose medium (Effelsberg et al., 2016). Upon shift from glucose to oleate medium, the cytosolic malate synthases Mls1p and Mls2p as well as Gto1p are transported into peroxisomes with the aid of Pex9p. Noteworthy, import of these enzymes can also be facilitated by the constitutively expressed Pex5p. Both PTS1 receptors interact with the peroxisomal docking protein Pex14p (Effelsberg et al., 2016). There is not much known about further processing of Pex9p at the peroxisomal membrane.

Here, we mapped the Pex9p-mediated protein import pathway by analyzing Pex9p-dependent protein import in various *pex* mutants devoid of Pex5p. The results suggest that Pex9p is using the same docking- and export apparatus as Pex5p, including ubiquitin-ligases and AAA+-peroxins. Pex9p differs significantly by its short lifetime when compared with Pex5p. Our data suggest that the rapid proteolytic breakdown of Pex9p is initiated by Pex4p-dependent ubiquitination at the peroxisomal membrane.

## Methods

### Strains and Media

The *S. cerevisiae* strains used in this study are listed in Supplementary Table 1. Cells were grown at 30°C in YPD (2% glucose, 2% peptone, 1% yeast extract) or selective media (0.5% ammonium sulfate, 0.17% yeast nitrogen base without amino acids, adjusted to pH 6.0) containing either 0.3% glucose (YNBG) or 0.1% glucose, 0.1% oleic acid, 0.05% Tween-40, and 0.1% yeast extract (YNBGO). Nutritional supplements were added when needed.

### Fixation of Yeast Cells for Fluorescence Microscopy

Cells transformed with GFP fused to PTS1 signal of Pcs60p (pLDC14) or Mls1p (pDE07), with additional Pex9p-ProtA (pMAR28) or Pex9C6A-ProtA (pMAR29) for complementation studies, were grown in YNBGO medium for 16 h. Formaldehyde was added to a final concentration of 3.7% and the culture was incubated for 10 min at RT with gentle agitation. After sedimentation (13.000 rpm, 1 min), cells were resuspended in 0.1M KH_2_PO_4_ (pH 6.8) supplemented with 3.7% formaldehyde. The cells were incubated for 1 h at RT with gentle agitation, sedimented, resuspended in 0.1M KH_2_PO_4_ (pH 6.8), containing 1% ethanolamine and incubated for further 10 min as indicated above. Afterward, cells were washed twice with PBS (pH 7.5). Finally, the cells were resuspended in a small volume of PBS, subjected to slides, and sealed with a cover slip.

### Fluorescence Microscopy

For wide-field fluorescence imaging, a Zeiss Axioskop 50 fluorescence microscope (Zeiss) was used. A Princeton Instruments 1300Y digital camera was used for image acquisition. GFP fluorescence was visualized using a 450–490 nm band pass excitation filter, a 510 nm dichromatic mirror, and a 515–565 nm band pass emission filter (Effelsberg et al., 2016).

### Cycloheximide Chase

Samples were taken from cells grown in YNGBO medium for 12 h (0 h) before either cycloheximide (final concentration 0.1 mg/ml, dissolved in DMSO) or only DMSO was added to the cultures. Cultures were grown at 30°C and samples were taken at the indicated time points. To generate total protein samples, a modified protocol from Yaffe and Schatz (1984) was utilized. Here, 300 μl samples of cells were prepared with an OD600 of 3.0, to which 100 μl 50% (w/v) TCA were added. The samples were frozen for at least 30 min at −68°C, thawed, and centrifuged for 15 min at 13.000 rpm and 4°C. The pellet was washed with ice-cold 80% acetone and subsequently centrifuged for 10 min at 13.000 rpm and 4°C. The supernatant was removed and the pellet dried at RT. In 80 μl 1% SDS/0.1M NaOH, the pellet was resuspended before 20 μl of 5x SDS-PAGE sample buffer were added.

### Steady State Level Analysis of Pex9p-ProtA and Pex9C6A-ProtA

Cells deficient in both Pex5p and Pex9p transformed with GFP-Mls1p in addition to Pex9p fused to ProteinA under its endogenous promoter (pMAR28) or a variant in which the conserved cysteine (Cys6) was substituted to alanine (pMAR29) were grown in YNBGO medium for 12 h. Of these, total protein samples were generated as described above.

## Results

### Pex9p- and Pex5p-Mediated Protein Import Depend on the Same Set of Peroxins at the Peroxisomal Membrane

**FIGURE 1:**
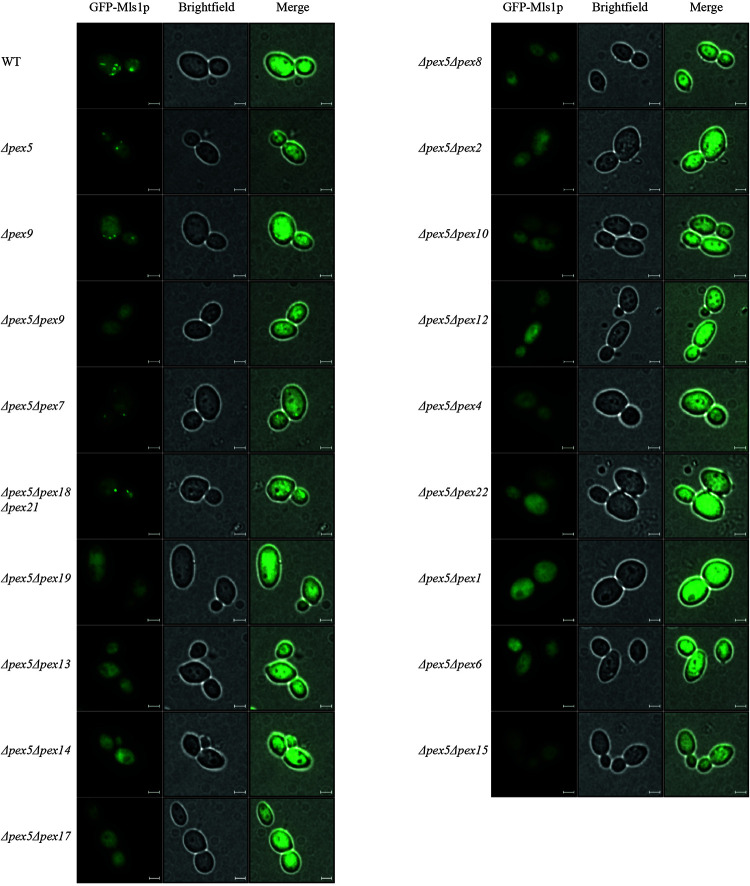
Dependence of Pex9p-related protein import on known components of the peroxisomal protein import machinery. Wild-type (WT) cells and yeast mutants deleted in one or more PEX genes (Δ*pex*X) were transformed with expression plasmids encoding GFP-Mls1p and grown for 16 h on a medium containing oleic acid. Fluorescence microscopy analysis of wild-type and single deletion mutants Δ*pex5* and Δ*pex9* exhibited a punctate pattern indicating peroxisomal association of the fusion protein GFP-Mls1p. With the exception of strains lacking the PTS2 (co-)receptors, Pex9p-dependent peroxisomal import is abolished in all tested double and triple deletion strains as indicated by diffuse cytosolic staining without puncta. Scale bars, 2 μm.

Pex9p was shown to associate with the peroxisomal membrane under oleate-inducing conditions (Effelsberg et al., 2016). To analyze further processing of Pex9p at the membrane, we investigated which peroxins are required for the Pex9p-dependent import pathway. To this end, several double deletion strains deficient in PEX5 and another PEX-gene of interest were analyzed for functional Pex9p-dependent import of GFP-Mls1p and the Pex5p-dependent import of GFP-PTS1 into peroxisomes. The Pex5p-cargo GFP-PTS1 was expressed from a plasmid and its peroxisomal import into oleic acid-induced cells was analyzed by fluorescence microscopy. As expected, a punctate fluorescence pattern indicative for functional import of GFP-PTS1 was observed in wild-type and PEX9-deleted cells. All other mutant strains showed no peroxisome import of the PTS1-marker protein as indicated by the diffuse cytosolic background (Supplementary Figure 1). The same mutant strains were transformed with a plasmid expressing the Pex9p-cargo GFP-Mls1p and subjected to fluorescence microscopy under oleate-inducing conditions (Figure 1). While the double mutant Δpex5Δpex9 showed no import of GFP-Mls1p, the single deletion mutant Δpex5 and all mutants affected in the PTS2 import pathway (Δpex5Δpex7 and Δpex5Δpex18Δpex21) exhibited discrete puncta, indicating that these peroxins are dispensable for GFP-Mls1p targeting to peroxisomes. The peroxisomal nature of the puncta is confirmed by the absence and thus cytosolic mislocalization of GFP-Mls1p in the peroxisome-depleted Δpex5Δpex19 strain (Hettema et al., 2000). No puncta were also detected in all other combinations of deleted genes, indicative for an import defect that results in a cytosolic mislocalization of the marker protein (Figure 1). Thus, the peroxisomal protein import mediated by Pex9p does not depend on the presence of Pex5p or constituents of the PTS2 import pathway but requires all known membrane-bound peroxins, which are also essential for Pex5p cycling. This involves the receptor docking complex consisting of Pex14p, Pex13p, and Pex17p as well as Pex8p, which is required for the association of the exportomer with the Pex5p docking/translocation complex. The Pex5p-ubiquitination machinery at the peroxisomal membrane consisting of the three ubiquitin E3-ligases Pex2p, Pex10p, and Pex12p, the E2-ligase Pex4p and its membrane anchor Pex22p are also required for Pex9p-facilitated import. Finally, the Pex9p-depended transport also depends on the AAA+ peroxins Pex1p and Pex6p (Figure 1). The peroxisomal association of Pex1p/Pex6p-complex is important as suggested by the requirement of Pex15p, which serves as a membrane anchor for these AAA+ peroxins. These findings suggest that Pex9p is released from the membrane like Pex5p in a Ubiquitin- and ATP-dependent manner.

### Pex9p Is Rapidly Degraded on Oleate-Containing Medium

**FIGURE 2:**
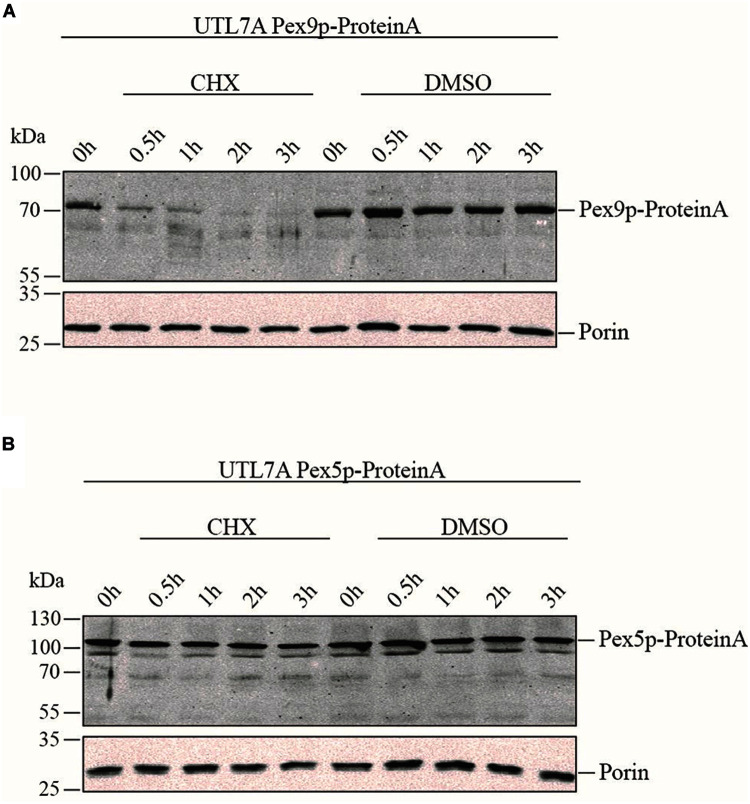
Rapid turnover rate of Pex9p under oleate-inducing conditions. Yeast UTL7A cells expressing ProteinA-tagged Pex9p **(A)** or Pex5p **(B)** under control of their own promotors were grown in YNBGO medium for 12 h. At this time point (0 h), 0.1 mg/ml cycloheximide (CHX) in DMSO (1%) was added to the medium and growth was continued. At indicated time points (0.5, 1, 2, and 3 h) samples were taken and analyzed by immunoblotting using polyclonal antibodies against ProteinA and mitochondrial Porin as loading control. To demonstrate that the steady state levels of proteins correlate with proteolytic degradation rather than new synthesis, yeast cells were incubated with equal volumes of DMSO without CHX (DMSO) and analyzed in the same way.

To compare the life-span of Pex9p and Pex5p, the strains UTL7A Pex9p-ProteinA expressing genomically tagged Pex9p under the control of its own promoter (Effelsberg et al., 2016) and the corresponding UTL7A Pex5p-ProteinA were grown under oleic acid inducing conditions in the absence and presence of the protein synthesis inhibitor cycloheximide (CHX). The steady-state level of both proteins was monitored over time by immunoblot analysis. Pex9p-ProteinA was degraded within 30 min, whereas, in absence of CHX, the initial protein level was maintained over the 3 h of the experiment (Figure 2A). These results indicate that Pex9p-ProteinA is rapidly degraded. Notably, the rapid proteolytic turnover is not a feature for Pex5p-ProteinA, which remained stable in the presence of CHX (Figure 2B).

### The Conserved Cysteine6 in Pex9p Is Critical for Its Functionality

**FIGURE 3:**
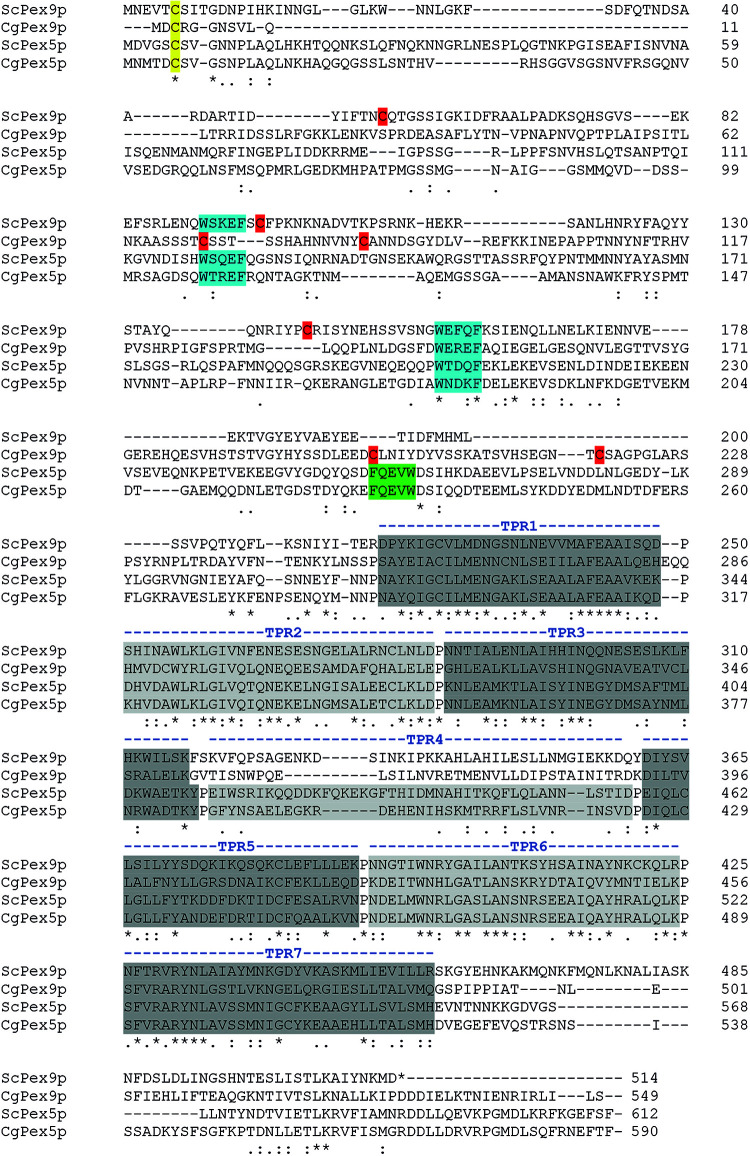
Identification of Pex9p from the pathogenic yeast *Candida glabrata*. A standard NCBI Protein BLAST search with fungal genomes (www.yeastgenome.org) with ScPex9p or ScPex5p as query identified two homologs in the pathogenic yeast *C. glabrata*. Using CLUSTAL O (1.2.4) multiple sequence alignment the unnamed protein product CAG61665.1 (CgPex5p) showed highest sequence identity (>49%) with ScPex5p while the open reading frame CAG61076.1 (CgPex9p) displayed identities of around 27% with ScPex5 and CAG61665.1 (CgPex5p) and 23% identity with ScPex9p. All identical amino acids are highlighted below the alignment lines by stars while conserved amino acids are marked by colons (strong conservation) and single dots (weak conservation). The regions with highest similarities of all PTS1 receptors and candidates comprise the TPR-domains within the C-terminal half (gray), WxxxF/Y motifs (blue) and the region around the conserved cysteine close to the N-terminus (yellow), which provides a site for monoubiquitination. Other Pex5p-specific sequence attributes like a conserved TPR4-domain, an inverted WxxxF/Y-motif (FQEVW) as part of the Pex14p-binding site as well as absence of cysteines (red) except the conserved one (yellow) in the N-terminal part of the receptor, were not found within the CAG61076.1 sequence. These findings support our conclusion that CAG61076.1 is an ortholog of Pex9p of *Saccharomyces cerevisiae*.

**FIGURE 4:**
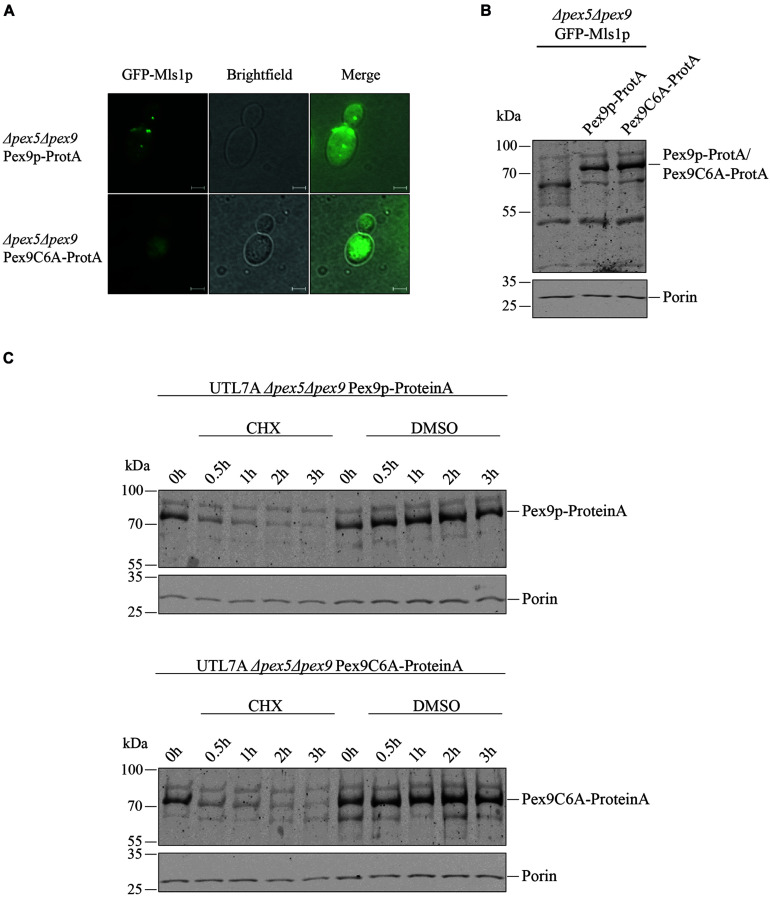
Cys6 of Pex9p is critical for protein import into peroxisomes. **(A)** UTL7A cells deficient in both Pex5p and Pex9p transformed with plasmids encoding GFP-Mls1p and a ProteinA (ProtA) fusion protein of either Pex9p or a Pex9C6A variant were grown for 16 h on a medium containing oleic acid and inspected by fluorescence microscopy. Expression of Pex9C6A could not complement the GFP-Mls1p localization defect to the cytosol while expression of the non-mutated Pex9p resulted in typical peroxisomal punctate labeling. Scale bars, 2 μm. **(B)** Steady-state level of both ProteinA fusion constructs was analyzed by immunoblotting using polyclonal anti ProteinA antibodies and anti Porin antibodies for loading control. **(C)** Stability of Pex9C6A-ProteinA in comparison to Pex9p-ProteinA was assessed by CHX chase as indicated in the legend of Figure 2. Here, no major differences in degradation rate depending on Cys6 could be detected.

Even though we found that the same import machinery is required for both PTS1 receptors, it was not yet clear whether Pex9p really undergoes a cyclic processing as Pex5p does. A checkpoint in Pex5p recycling is monoubiquitination of a unique cysteine close to the N-terminus (Williams et al., 2007; Schwartzkopff et al., 2015). Sequence alignments revealed that the position and sequence environment of the specific cysteine residue is conserved between Pex5p and Pex9p in *S. cerevisiae* as well as in the pathogenic yeast *Candida glabrata* (Figure 3). By site-directed mutagenesis, we generated a ProteinA tagged variant of Pex9p, in which the conserved cysteine at position is substituted with an alanine under control of its own promotor. We assessed the functionality of Pex9C6A-ProteinA by fluorescence microscopy, utilizing a strain deficient in both Pex5p and Pex9p and expressing GFP-Mls1p. We found that Pex9C6A-ProteinA, in contrast to the unmodified Pex9p-ProteinA, could not rescue import of GFP-Mls1p into peroxisomes (Figure 4A), even though it was expressed to the same amount as the unmodified version (Figures 4B,C). In addition, no significant differences regarding the degradation rate of Pex9p-ProteinA and Pex9C6A-ProteinA could be detected in cycloheximide chase experiments (Figure 4C).

### Discussion

The import cycle of the import receptor Pex5p requires the sequential action of several peroxins that contribute to receptor docking, pore formation, ubiquitination and ATP-dependent export and recycling. Here, we showed that the novel PTS1-receptor Pex9p requires the same set of membrane-associated peroxins to facilitate protein import into peroxisomes as Pex5p and PTS2 receptors. In analogy to the Pex5p-dependent protein import into peroxisomes, a complete picture of the Pex9p-dependent import into peroxisomes can now be postulated. According to this model, the Pex9p-cargo complex docks at a membrane complex consisting of Pex13p, Pex14p and Pex17p. High binding affinity between Pex9p and Pex14p was shown previously, while the other two proteins of this complex displayed no visible interaction (Effelsberg et al., 2016). The strength of Pex14p binding and conservation of Pex14p-binding motifs in the Pex9p sequence suggest that Pex14p fulfils a similar role in both PTS1-dependent import routes. In such a scenario, Pex14p would function as initial docking protein for the Pex9p receptor cargo complex and would contribute to the formation of a transient import pore, which is supposed to mediate cargo translocation across the peroxisomal membrane (Meinecke et al., 2010). Also, the peroxins of the export complex comprising the ubiquitination machinery (Pex2p, Pex4p, Pex10p, Pex12p, and Pex22p) and the AAA^+^-complex (Pex1p, Pex6p, and Pex15p) are required for the Pex9p-dependent peroxisomal targeting of Mls1p (Figure 1). This result suggests that, after dissociation of the receptor-cargo complexes, Pex9p, like the other import receptors, uses a complex network of protein-protein interactions that finally results in the release of the receptor from the membrane. For Pex5p it is known that, at the end of the import process, the protein is monoubiquitinated at a conserved cysteine, which functions as export signal for the ATP- and Pex1p/Pex6p-dependent dislocation of the receptor from the membrane to the cytosol (Platta et al., 2005, 2007; Williams et al., 2007). The requirement for the ubiquitin-conjugating Pex4p and the E3-ligases Pex2p, Pex10p, and Pex12p suggests that Pex9p is also ubiquitinated. In support of this assumption, the conserved cysteine that is monoubiquitinated in Pex5p, Pex18p, Pex20p, and Pex21p is also conserved in the Pex9p-sequence of *S. cerevisiae* and in the newly identified Pex9p sequence of the pathogenic *C. glabrata* (Figure 3). Substitution of this cysteine in Pex5p, Pex18p, and Pex20p by residues which are normally not ubiquitinated impairs functional protein import (Leon and Subramani, 2007; Williams et al., 2007; Hensel et al., 2011). Accordingly, expression of Pex9C6A cannot rescue the import defect of PEX9-deleted yeast cells. All in all, the results obtained with the genetic screen and the complementation analysis basically reveal no major differences between the processing of Pex9p and Pex5p at the peroxisomal membrane.

Although the import pathways of Pex5p and Pex9p utilize the same components, the fate of the extracted PTS1 receptors is different. Where Pex5p is mostly recycled to enable further rounds of import, the bulk of Pex9p is rapidly degraded under oleate-induced conditions. This is underlined by different turnover rates. While the half-life of constitutively expressed Pex5p was estimated to be 2.5 h on glucose medium (Christiano et al., 2014), our results suggest a shorter lifetime of Pex9p, which is below 30 min in oleate medium (Figure 2).

Interestingly, the turnover rate of biologically inactive Pex9C6A in oleate grown cells is the same as for wild type. This suggests that Pex9p degradation occurs independently from functional import. The mechanisms underlying the fast degradation process of Pex9p are not known. However, other peroxisomal receptor variants which are impaired in monoubiquitination and thereby, get stuck in the import pathway are degraded by proteasomes in a kind of quality control (Kiel et al., 2005; Leon and Subramani, 2007; Platta et al., 2007; Schwartzkopff et al., 2015). In accordance with the similarities between peroxisomal import pathways, it seems likely that proteasomal breakdown determines the short lifetime of Pex9p.

The rapid turnover rate of Pex9p correlates with transcriptional regulation and function of the PTS1- receptor. Pex9p is expressed under specific growth conditions and highly selective for a limited number of PTS1 cargo proteins. The known cargo enzymes, malate synthases Mls1p and Mls2p, are catalytically fully active in the cytosol and required for metabolism in cells grown in glucose, ethanol or acetate. Upon shift on oleate medium, the enzymes are transported into peroxisomes where fatty acids are oxidized to metabolites which are further converted by malate synthases. In other words, the activity of Pex9p helps to optimize yeast metabolism during growth on fatty acid as carbon source. The short lifetime of Pex9p combined with a carbon source-dependent transcriptional regulation enables the yeast to adapt the intracellular localization of malate synthases as a fast response to environmental changes, like a shift from oleate to other carbon sources. The efficacy of a “switchable” Pex9p-dependent import pathway is ensured since this route relies on an already existing peroxin network at the peroxisomal membrane. By sequence comparison, we identified a putative ortholog of the alternative PTS1-receptor Pex9p in a different yeast species, the pathogenic fungus *C. glabrata*. During its parasitic life, *C. glabrata* can persist and replicate within macrophages (Seider et al., 2014). In this microenvironment, the fungus must undergo fast metabolic adaptation due to the deficiency of glucose, the most dominant carbon source in the bloodstream. It has been reported (Rai et al., 2012) that, under these conditions, fatty acid oxidation and the glyoxylate cycle become major metabolic pathways to provide cellular building blocks for survival, growth and proliferation within the monocytes (Chew et al., 2019a,b). It is tempting to speculate that the rapid metabolic reprogramming is accompanied by a localization shift of metabolic enzymes from cytosol into peroxisomes relying on the Pex9p homolog in the human pathogen.

The differences in the regulation of Pex5p and Pex9p abundance show parallels to PTS2 import, which engages two paralogous co-receptors, Pex18p and Pex21p. These proteins were identified and characterized in *S. cerevisiae*, but have also been detected in *C. glabrata* (Schliebs and Kunau, 2006). Like Pex9p, Pex18p is also oleate-inducible and highly selective toward cargo-proteins. The fatty acid degrading enzyme thiolase is the only known cargo protein imported by the Pex18p-dependent pathway. In contrast, the functionally redundant Pex21p has a broader selectivity of PTS2-cargo proteins, which are translocated into peroxisomes also under non-oleate growth conditions. In this context, it is interesting that like Pex9p, Pex18p is an extremely short-lived protein with a half-life of below 30 min (Purdue and Lazarow, 2001). With respect to these similarities in gene expression and turnover of PTS1 and PTS2 receptors, the peroxisomal import pathways in baker’s yeast fall into two distinct functional classes. While Pex5p and Pex21p facilitate constitutive import pathways for peroxisomal protein import, the strictly regulated receptors Pex9p and Pex18p improve the efficacy of the import machinery under specialized conditions.

## Data Availability Statement

All datasets presented in this study are included in the article/Supplementary Material.

## Author Contributions

MR, RE, and WS conceived and planned the experiments, contributed to the interpretation of the results, and writing of the manuscript. MR carried out the experiments and analyzed the data. WS performed the sequence analyses. All authors contributed to the article and approved the submitted version.

## Conflict of Interest

The authors declare that the research was conducted in the absence of any commercial or financial relationships that could be construed as a potential conflict of interest.
