# Supplementary figures and images for: Membrane Processing and Steady-State Regulation of the Alternative Peroxisomal Import Receptor Pex9p
Source: Front Cell Dev Biol. 2020 Oct 22;8:566321. doi: 10.3389/fcell.2020.566321 (PMC7642143; doi:10.3389/fcell.2020.566321)

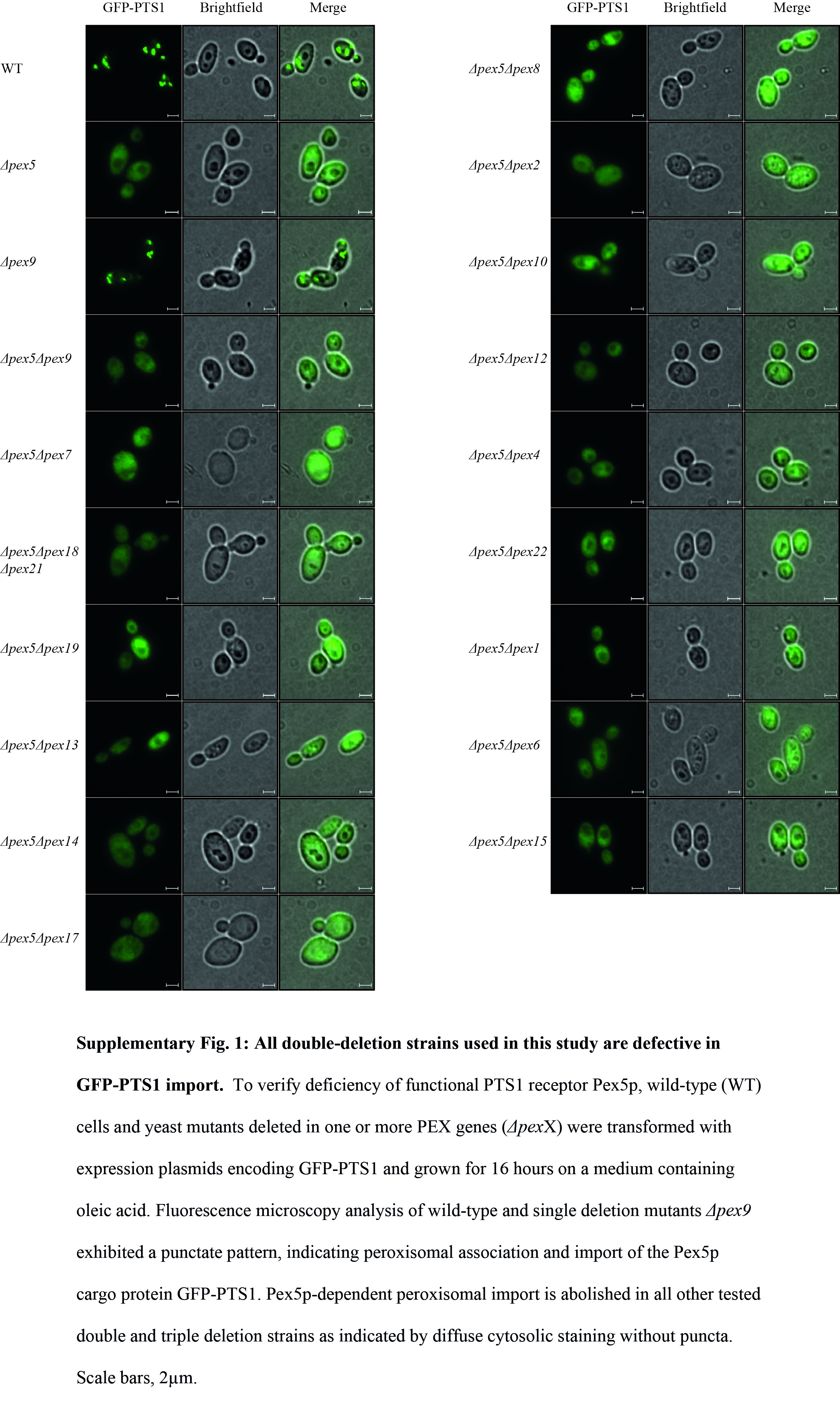

Supplement: Supplementary file 1 [file Image_1.JPEG]
