# Supplementary material for: Membrane Processing and Steady-State Regulation of the Alternative Peroxisomal Import Receptor Pex9p
Source: Front Cell Dev Biol. 2020 Oct 22;8:566321. doi: 10.3389/fcell.2020.566321 (PMC7642143; doi:10.3389/fcell.2020.566321)
Supplement: Supplementary file 2 [file Table_1.DOCX]

**Supplementary Table 1:** *Saccharomyces cerevisiae* strains utilized in this study

| Name | Genotype | Source |
| --- | --- | --- |
| UTL7A wild-type | *MATα; ura3-52; trp1; leu2-3/112* | (Erdmann et al., 1989) |
| UTL7A *pex5Δ* | UTL7A *pex5::loxP* | (Girzalsky et al., 1999) |
| UTL7A *pex5Δpex1Δ* | UTL7A *pex1::loxP removed, pex5::kanMX4* | (Platta et al., 2007) |
| UTL7A *pex5Δpex2Δ* | UTL7A *pex2::LEU, pex5::loxP removed* | This study |
| UTL7A *pex5Δpex4Δ* | UTL7A *pex4::LEU, pex5::loxP removed* | This study |
| UTL7A *pex5Δpex6Δ* | UTL7A *pex5::loxP removed, pex6::loxP removed* | This study |
| UTL7A *pex5Δpex7Δ* | UTL7A *pex5::kanMX4, pex7::LEU* | This study |
| UTL7A *pex5Δpex8Δ*Pex18-TEV-ProteinA | UTL7A *pex5::loxP removed, pex8::loxP removed, Pex18-TEV-Prot.A* | This study |
| UTL7A *pex5Δpex9Δ* | UTL7A *pex5::loxP; pex9::KanMX6* | (Effelsberg et al., 2016) |
| UTL7A *pex5Δpex10Δ* | UTL7A *pex5::loxP removed, pex10::loxP removed* | This study |
| UTL7A *pex5Δpex12Δ* | UTL7A *pex5::CreloxP, pex12::LEU* | This study |
| UTL7A *pex5Δpex13Δ* | UTL7A *pex5::loxP removed, pex13::loxP removed* | This study |
| UTL7A *pex5Δpex14Δ* | UTL7A *pex5::loxP removed, pex14::loxP removed* | This study |
| UTL7A *pex5Δpex15Δ* | UTL7A *pex5::lox removed, pex15::lox removed* | This study |
| UTL7A *pex5Δpex17Δ* | UTL7A *pex5::loxP removed, pex17::LEU* | This study |
| UTL7A *pex5Δpex18Δpex21Δ* | UTL7A *pex5::CreloxP, pex18::lox removed, pex21::lox removed* | This study |
| UTL7A *pex5Δpex19Δ* | UTL7A *pex5::loxP removed, pex19::CreloxP* | This study |
| UTL7A *pex5Δpex22Δ* | UTL7A *pex5::loxP removed, pex22::CreloxP* | This study |
| UTL7A *pex9Δ* | UTL7A *pex9::loxP* | (Effelsberg et al., 2016) |
| UTL7A Pex5-Prot.A | UTL7A ::*PEX5-ProtA*-KanMX4 | (Kiel et al., 2005) |
| UTL7A Pex9-TEV-ProteinA | UTL7A *PEX9-TEV-ProteinA-kanMX6* | (Effelsberg et al., 2016) |

**Supplementary Table 2:** Plasmids used in this study

| Name | Description | Source | Primer sequence |
| --- | --- | --- | --- |
| pDE07 | pUG36-GFP-PCS60(531-543) | (Effelsberg et al., 2016) |  |
| pLDC14 | pRS415-PEX9prom-PEX9-TEV-ProtA | This study |  |
| pMAR28 | pRS415-PEX9prom-Pex9C6A-TEV-ProtA | This study | RE6837: cgctcactggccgtcg  RE7262: CGTACGCTGCAGGTCGAC  RE7263: cgacggccagtgagcgGAATTG  TCTTGATTGGGTGCTTATGCTG  RE7264: GTCGACCTGCAGCGTACGATCC  ATTTTGTTATAAATAGCTTTTAA  GGTTG |
| pMAR29 | pUG36-GFP-PCS60(531-543) | This study | RE7307: GATGAACGAAGTTACTGCTTCC  ATAACTGGTGAC  RE7308: GTCACCAGTTATGGAAGCAGTA  ACTTCGTTCATC |

**References**

Effelsberg, D., Cruz-Zaragoza, L.D., Schliebs, W., and Erdmann, R. (2016). Pex9p is a new yeast peroxisomal import receptor for PTS1-containing proteins. *J Cell Sci* 129(21)**,** 4057-4066. doi: 10.1242/jcs.195271.

Erdmann, R., Veenhuis, M., Mertens, D., and Kunau, W.-H. (1989). Isolation of peroxisome-deficient mutants of *Saccharomyces cerevisiae*. *Proc. Natl. Acad. Sci. USA,* 86**,** 2432-2436.

Girzalsky, W., Rehling , P., Stein, K., Kipper, J., Blank, L., Kunau, W.-H., et al. (1999). Involvement of Pex13p in Pex14p localization and peroxisomal targeting signal 2 dependent protein import into peroxisomes. *J. Cell Biol.* 144(6)**,** 1151-1162.

Kiel, J.A., Emmrich, K., Meyer, H.E., and Kunau, W.H. (2005). Ubiquitination of the peroxisomal targeting signal type 1 receptor, Pex5p, suggests the presence of a quality control mechanism during peroxisomal matrix protein import. *J Biol Chem* 280(3)**,** 1921-1930.

Platta, H.W., El Magraoui, F., Schlee, D., Grunau, S., Girzalsky, W., and Erdmann, R. (2007). Ubiquitination of the peroxisomal import receptor Pex5p is required for its recycling. *J Cell Biol* 177(2)**,** 197-204.
